# Construction and validation of a morbidity index based on the International Classification of Primary Care

**DOI:** 10.1080/02813432.2022.2097617

**Published:** 2022-07-13

**Authors:** Hogne Sandvik, Sabine Ruths, Steinar Hunskaar, Jesper Blinkenberg, Øystein Hetlevik

**Affiliations:** a National Centre for Emergency Primary Health Care, NORCE Norwegian Research Centre, Bergen, Norway; b Department of Global Public Health and Primary Care, University of Bergen, Bergen, Norway; c Research Unit for General Practice, NORCE Norwegian Research Centre, Bergen, Norway

**Keywords:** Administrative claims, general practice, international classification of disease codes, morbidity, mortality, primary health care

## Abstract

**Objectives:**

In epidemiological studies it is often necessary to describe morbidity. The aim of the present study is to construct and validate a morbidity index based on the International Classification of Primary Care (ICPC-2).

**Design and Setting:**

This is a cohort study based on linked data from national registries. An ICPC morbidity index was constructed based on a list of longstanding health problems in earlier published Scottish data from general practice and adapted to diagnostic ICPC-2 codes recorded in Norwegian general practice 2015 − 2017.

**Subjects:**

The index was constructed among Norwegian born people only (*N* = 4 509 382) and validated in a different population, foreign-born people living in Norway (*N* = 959 496).

**Main outcome measures:**

Predictive ability for death in 2018 in these populations was compared with the Charlson index. Multiple logistic regression was used to identify morbidities with the highest odds ratios (OR) for death and predictive ability for different combinations of morbidities was estimated by the area under receiver operating characteristic curves (AUC).

**Results:**

An index based on 18 morbidities was found to be optimal, predicting mortality with an AUC of 0.78, slightly better than the Charlson index (AUC 0.77). External validation in a foreign-born population yielded an AUC of 0.76 for the ICPC morbidity index and 0.77 for the Charlson index.

**Conclusions:**

The ICPC morbidity index performs equal to the Charlson index and can be recommended for use in data materials collected in primary health care.

**Key points**
- This is the first morbidity index based on the International Classification of Primary Care, 2^nd^ edition (ICPC-2)
- It predicted mortality equal to the Charlson index and validated acceptably in a different population
- The ICPC morbidity index can be used as an adjustment variable in epidemiological research in primary care databases

## Introduction

In epidemiological studies of different health outcomes there is often a need to describe morbidity and comorbidity among patients or in a population. The outcomes of interest in analyses that need such tools could be mortality, effect of treatment, use of health care or health care cost. Many morbidity indices have been developed in recent decades, with different purposes [1–3]. The most widely used tool is the Charlson index which was originally developed in 1987 to account for comorbid conditions that could influence mortality among patients admitted to a medical service at a New York hospital [4].

The Charlson index was later translated into International Classification of Diseases (ICD) codes suited for registry-based epidemiological research [5,6]. There has also been a series of adaptations with different selections of diagnoses, and different weighting of the diagnoses. The Royal College of Surgeons’ version from 2017 includes 14 disease categories without weighting, suitable for use with data from administrative databases or registries, and it has performed well as a predictor of mortality [5]. With an increasing availability of large datasets in administrative and research databases, morbidity indices will play an important role as adjusting variables in statistical analyses.

The Charlson index was developed among hospitalised patients and may not be equally well suited for primary care research. An important limitation is the lack of psychiatric diagnoses in this index. Therefore, morbidity indices developed in primary care are needed. Some versions have adapted the Charlson index with codes used in primary care, such as the Read codes used in UK primary care and primary care databases [7,8].

A study comparing a Charlson index based on data from secondary care with data from primary care showed similar predictive ability regarding mortality [9]. However, the selection of disease categories was mainly the same as the selection used in the original Charlson index. An index constructed with a new selection of diagnoses based on primary care data in the UK explained mortality at practice level better than the Charlson index [10].

Although the original Charlson index was developed with mortality as an outcome, it was later adapted for a variety of purposes, such as to assess burden of disease and predicting costs and hospitalization [11–13]. However, indices often perform differently depending on the outcome of interest and should therefore probably be developed for a specific outcome [1,2]. According to a systematic review, indices based on diagnoses alone seem best at predicting mortality, and, moreover including information about prescriptions can improve the predictive ability regarding the use of health care [3].

A systematic search of the literature has revealed no indices predicting mortality based on the International Classification of Primary Care, 2nd edition (ICPC-2) [14]. ICPC-2 is a classification system developed for primary care by WONCA (World Organization of Family Doctors) and is a part of the WHO family of international classifications in use in several countries, including Norway.

The aim of the present study is to develop and validate an ICPC morbidity index to predict mortality using nation-wide registry data in Norway.

## Methods

### Design and data sources

This is a cohort study based on linked data from national health and population registries, 2015 − 2018. Predictor (explanatory) variables were collected from 2015 − 2017 and outcome variables from 2018. In Norway, all citizens including foreigners staying for more than six months, are given a unique identification number. This number is used in many official records and makes it possible to link data from these registries at the individual patient level.

Statistics Norway (SSB) provided demographic information (gender, country of birth, age and death during 2018). Country of birth was recoded into Norwegian-born or foreign-born.

Primary care doctors send compensation claims to the Norwegian Health Economics Administration (HELFO) for all patient contacts. This goes for both regular general practitioners and out-of-hours doctors in the municipalities. Compensation claims include one or more diagnoses according to ICPC-2 [14]. For this study we included ICPC-2 diagnostic codes recorded for all types of contact during the years 2015 − 2017. These diagnoses were used when constructing the new ICPC morbidity index.

The Norwegian Patient Registry (NPR) provided information about all patient contacts with specialist health care. All diagnostic codes (ICD-10) recorded during the years 2015 − 2017, either outpatient or inpatient, were used to calculate the Charlson index.

### Analysis strategy

Development of the ICPC morbidity index was performed among Norwegian born people only (*N* = 4 509 382). The ability of the index to predict death during 2018 was compared with the Charlson index serving as a gold standard. For validation, a similar analysis was performed in a different population, namely foreign-born people living in Norway (*N* = 959 496).

### Construction of the ICPC morbidity index

In 2012 Karen Barnett *et al.* published a paper on the distribution of multimorbidity in general practice in Scotland [15]. Based on literature research and national databases they established a list of 40 long-term conditions. In 2020 Payne *et al.* found that the Cambridge Multimorbidity Score, based on the same list, also predicted mortality [16]. We chose this established list of longstanding conditions as basis for our analyses.

The list of health conditions from Barnett *et al.* was defined by one or more Read codes and in some cases also by drug treatment. We created a list of 38 morbidities defined by corresponding ICPC-2 codes (Table 1), and made the following adaptations: Omitted two of the 40 morbidities, bronchiectasis, because no corresponding ICPC-2 code exists; and treated constipation, because primary care databases do not necessarily contain information on drug prescription. Furthermore, we defined painful conditions as specific long-term musculoskeletal and neurological morbidities that usually include a substantial symptom burden. Similar adaptions were also used for other morbidity groups, such as defining them solely with diagnostic codes and no knowledge of prescriptions.

**Table 1.** Application of ICPC-2 diagnostic codes to 38 morbidities collected from a database of 1 751 841 people registered with 314 medical practices in Scotland [15]. Odds ratio (OR) for death in 2018, based on the same ICPC-2 diagnoses recorded in Norway during 2015–2017. All 38 morbidities were included in a single multivariable logistic regression analysis, adjusted for gender and age. The 18 morbidities marked in bold were included in the final ICPC morbidity index. Data material: Norwegian born (N = 4 509 382).

Morbidities 2015–2017	ICPC-2 codes	Number of patients	Death 2018		
			Regression coefficient (B)	OR	95 % CI
**Cancer**	**A79, B72, B73, B74, D74, D75, D76, D77, L71, N74, R84, R85, T71, U75. U76, U77, W72, X75, X76, X77, Y77, Y78**	**119 824**	**1.15**	**3.16**	**3.08 − 3.25**
**Viral hepatitis**	**D72**	**6 515**	**0.80**	**2.23**	**1.81 − 2.75**
Dyspepsia	D84, D85, D86, D87	153 547	0.01	1.01	0.97 − 1.06
Diverticular disease of intestine	D92	24 825	−0.23	0.80	0.72 − 0.88
Irritable bowel disorder	D93	28 492	−0.47	0.63	0.51 − 0.76
Inflammatory bowel disease	D94	32 685	0.10	1.11	0.98 − 1.25
**Chronic liver disease**	**D97**	**18 412**	**0.63**	**1.88**	**1.70 − 2.07**
Glaucoma	F93	18 908	0.01	1.01	0.94 − 1.08
Blindness & low vision	F94	7 778	0.18	1.19	1.05 − 1.35
Hearing loss	H83, H84, H85, H86	48 094	−0.21	0.81	0.77 − 0.86
Coronary heart disease	K74, K75, K76	116 144	−0.05	0.95	0.92 − 0.98
**Heart failure**	**K77**	**39 279**	**0.86**	**2.37**	**2.29 − 2.46**
Atrial fibrillation	K78	96 002	0.16	1.17	1.13 − 1.21
Hypertension	K86, K87	527 280	−0.32	0.73	0.71 − 0.74
**Stroke & transient ischaemic attack**	**K89, K90**	**64 506**	**0.34**	**1.41**	**1.36 − 1.47**
**Peripheral vascular disease**	**K92**	**29 022**	**0.26**	**1.29**	**1.21 − 1.37**
Painful condition	L18, L83, L84, L85, L86, L89, L90, L91, L92, N90, N94, N95	878 845	−0.37	0.69	0.67 − 0.71
Rheumatoid arthritis, other inflammatory polyarthropathies & systematic connective tissue disorders	L88	61 630	0.16	1.18	1.11 − 1.25
**Multiple sclerosis**	**N86**	**11 901**	**0.79**	**2.21**	**1.88 − 2.59**
**Parkinson’s disease**	**N87**	**11 356**	**0.87**	**2.38**	**2.22 − 2.56**
**Epilepsy**	**N88**	**32 879**	**0.75**	**2.11**	**1.94 − 2.29**
Migraine	N89	120 858	−0.23	0.79	0.69 − 0.91
**Alcohol problems**	**P15**	**27 488**	**1.05**	**2.87**	**2.66 − 3.09**
**Other psychoactive substance misuse**	**P18, P19**	**34 167**	**1.36**	**3.88**	**3.50 − 4.30**
**Learning disability**	**P24, P85**	**26 392**	**1.11**	**3.04**	**2.62 − 3.52**
**Dementia**	**P70**	**31 374**	**1.09**	**2.96**	**2.86 − 3.06**
**Schizophrenia (and related nonorganic psychosis) or bipolar disorder**	**P72, P73, P98**	**46 734**	**0.75**	**2.12**	**1.96 − 2.29**
Anxiety & other neurotic, stress related & somatoform disorders	P74, P75, P82	131 149	0.22	1.24	1.17 − 1.32
Depression	P76	271 031	0.23	1.26	1.21 − 1.32
**Anorexia or bulimia**	**P86**	**4 394**	**0.91**	**2.48**	**1.43 − 4.29**
Sinusitis	R75	201 324	−0.42	0.66	0.60 − 0.71
**Chronic obstructive pulmonary disease**	**R95**	**91 012**	**0.77**	**2.17**	**2.10 − 2.24**
Asthma	R96	248 978	−0.10	0.91	0.86 − 0.95
Psoriasis or eczema	S86, S87, S88, S91	335 956	−0.09	0.91	0.87 − 0.95
Thyroid disorders	T85, T86	155 695	−0.13	0.88	0.84 − 0.92
**Diabetes**	**T89, T90**	**186 227**	**0.27**	**1.30**	**1.26 − 1.34**
**Chronic kidney disease**	**U88**	**3 167**	**0.72**	**2.05**	**1.64 − 2.55**
Prostate disorders	Y85	47 836	−0.28	0.76	0.71 − 0.80

Thereafter, we identified every patient recorded with one or more of these diagnostic codes in Norwegian primary care compensation claims during the years 2015 − 2017. Of the 38 morbidities, 18 were included in the final ICPC morbidity index based on their strength of association with mortality (described in the statistics section below).

### Statistics

A multivariable logistic regression analysis was performed using all 38 morbidities as predictors of death during 2018, adjusted for each other and for sex and age, but only including Norwegian-born individuals (Table 1). The morbidities with the highest odds ratios (OR) were included in the index.

The number of morbidities for each patient was categorised into four groups: zero, one, two and three or more. We explored the performance of different indices as predictors of mortality with 16 − 20 morbidities included. This was done by considering the number of patients and OR, as well as by receiving operating characteristic (ROC) curves with the area under curve (AUC) for each index. The index with the highest possible combination of many patients, a high OR, and a high AUC was chosen. It has been suggested that AUC (or C-statistics) values of 0.7 to 0.8 show acceptable discrimination, while values of 0.8 to 0.9 indicate excellent discrimination and values >0.9 outstanding discrimination [17].

As recommended by Steyerberg *et al.*, internal validation of the chosen 18-item index was done by bootstrapping analyses of OR and AUC with 1 000 repetitions [18]. Sensitivity analysis was performed by narrowing the predictor morbidities to those recorded during 2017 only. We also analysed a weighted index, multiplying each morbidity with the regression coefficient.

In a similar analysis OR and AUC were calculated for the Charlson index (2015 − 2017) as predictors of death during 2018. We then examined the performance of the ICPC morbidity index and Charlson index in a new population, namely foreign-born people living in Norway, again with death during 2018 as an outcome.

The analyses were carried out using SPSS version 27. Bootstrapping was performed with Stata version 16.

## Results

### Construction of index

OR for death for each of the 38 different morbidities are given in Table 1 and adjusted for all other morbidities, age and sex. Table 2 shows the number of patients, OR, and AUC for possible indices with 16 − 20 morbidities included. There was an inverse relationship between the number of patients included in the models and OR for each level of the index. The optimal compromise was found to be an index with 18 morbidities, which had the highest AUC (0.78, 95% CI 0.77–0.78).

**Table 2.** Predictability of alternative ICPC morbidity indices (different number of morbidities included) for death in 2018 among Norwegian born people living in Norway (*N* = 4 509 382). The table also includes a similar analysis of the Charlson index in the same population.

	N	OR	95 % CI
ICPC morbidity index with 16 morbidities			
1	405 528	3.23	3.15 − 3.30
2	64 734	5.93	5.73 − 6.13
3+	10 948	9.96	936 − 10.59
Sum	481 210	AUC: 0.76 (0.76 − 0.76)	
ICPC morbidity index with 17 morbidities			
1	505 092	2.73	2.66 − 2.80
2	95 176	4.98	4.83 − 5.14
3+	19 015	8.58	8.17 − 9.02
Sum	619 283	AUC: 0.77 (0.77 − 0.78)	
ICPC morbidity index with 18 morbidities			
1	512 531	2.68	2.61 − 2.74
2	101 196	4.81	4.66 − 4.96
3+	21 914	8.12	7.75 − 8.50
Sum	635 641	AUC: 0.78 (0.77 − 0.78)	
ICPC morbidity index with 19 morbidities			
1	659 346	2.49	2.43 − 2.55
2	141 596	4.52	4.39 − 4.66
3+	35 139	7.42	7.12 − 7.74
Sum	836 081	AUC: 0.77 (0.77 − 0.77)	
ICPC morbidity index with 20 morbidities			
1	681 603	2.43	2.37 − 2.49
2	172 544	4.28	4.15 − 4.41
3+	49 087	7.14	6.86 − 7.43
Sum	903 234	AUC: 0.76 (0.76 − 0.77)	
Charlson index			
1	452 152	1.85	1.80 − 1.90
2	104 851	4.19	4.07 − 4.32
3+	45 774	7.77	7.52 − 8.02
Sum	602 777	AUC: 0.77 (0.76 − 0.77)	

Odds ratio (OR) adjusted for gender and age. Multivariable logistic regression analyses with zero morbidities as reference category. AUC: Area under curve.

### Validation

**Figure 1.**
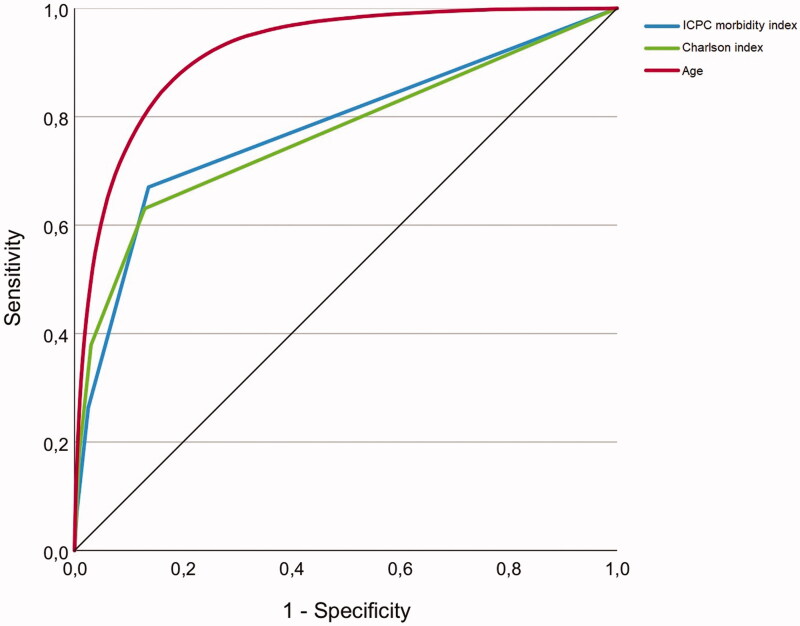
Receiver operating characteristic (ROC) curves for the ICPC morbidity index, the Charlson index and age as predictors of death in 2018 among Norwegian born people living in Norway (*N* = 4 509 382).

The Charlson index applied to the same population is also shown in Table 2. Compared with the 18-item ICPC morbidity index, the Charlson index revealed slightly lower OR and AUC. Figure 1 shows ROC curves for both indices and age.

Bootstrapping the multiple regression analysis for the 18-item index yielded the same point estimates, with a slightly wider confidence interval affecting only the second decimal (data not shown). Moreover, bootstrapping the AUC analysis did not change the results.

Weighting the index with the regression coefficients of the individual morbidities slightly increased the OR (2.69 (95% CI 2.62–2.75), 5.81 (5.62–6.01) and 9.18 (8.73–9.65) for 1, 2 and 3+ morbidities, respectively) and marginally reduced the AUC (0.77). Harvesting diagnoses only from 2017 resulted in slightly lower OR (2.42 (2.36–2.47), 4.60 (4.44–4.77), 7.84 (7.26–8.46) for 1, 2 and 3+ morbidities, respectively) and clearly lower AUC (0.71).

In Table 3 the ICPC morbidity index and Charlson index have been applied on a different population, namely foreign-born people living in Norway. The OR was higher in the foreign-born population than in the Norwegian-born population, both for the ICPC morbidity index and for the Charlson index. Again, the ICPC morbidity index demonstrated slightly higher ORs, while the AUC was slightly lower than for the Charlson index.

**Table 3.** Predictability of the ICPC morbidity index and the Charlson index for death among foreign-born people living in Norway (*N* = 959 496).

	N	OR	95 % CI
ICPC morbidity index			
1	69 216	3.37	3.04 − 3.72
2	7 818	7.68	6.59 − 8.95
3+	1 928	15.11	12.07 − 18.93
Sum	78 962	AUC: 0.76 (0.75 − 0.78)	
Charlson index			
1	54 408	2.57	2.28 − 2.89
2	9 673	6.93	6.08 − 7.89
3+	3 537	13.07	11.35 − 15.04
Sum	67 618	AUC: 0.77 (0.75 − 0.78)	

Odds ratio (OR) adjusted for gender and age. Multivariable logistic regression analyses with zero morbidities as reference category. AUC: Area under curve.

The AUC was slightly higher for males than for females, 0.79 (0.79–0.80) vs. 0.76 (0.76–0.77) for the ICPC morbidity index and 0.79 (0.79–0.79) vs. 0.75 (0.74–0.75) for the Charlson index. For age <40 years the AUC was 0.71(0.69–0.74) for the ICPC morbidity index and 0.60 (0.57–0.62) for the Charlson index. For age 40 − 69 years the AUC was 0.77 (0.77–0.78) and 0.76 (0.75–0.77) for the ICPC and Charlson index, respectively and for age ≥70 years the AUC was 0.66 (0.66–0.66) for the ICPC index and 0.66 (0.66–0.67) for the Carlson index.

### Final version

The complete ICPC morbidity index is shown in Table 4.

**Table 4.** The ICPC morbidity index comprises 18 morbidities with the following ICPC-2 diagnostic codes.

Morbidities	ICPC-2 codes
Cancer	A79, B72, B73, B74, D74, D75, D76, D77, L71, N74, R84, R85, T71, U75. U76, U77, W72, X75, X76, X77, Y77, Y78
Viral hepatitis	D72
Chronic liver disease	D97
Heart failure	K77
Stroke & transient ischaemic attack	K89, K90
Peripheral vascular disease	K92
Multiple sclerosis	N86
Parkinson’s disease	N87
Epilepsy	N88
Alcohol problems	P15
Other psychoactive substance misuse	P18, P19
Learning disability	P24, P85
Dementia	P70
Schizophrenia (and related nonorganic psychosis) or bipolar disorder	P72, P73, P98
Anorexia or bulimia	P86
Chronic obstructive pulmonary disease	R95
Diabetes	T89, T90
Chronic kidney disease	U88

## Discussion

The ICPC morbidity index predicted mortality equal to the Charlson index. It validated acceptably in a different population.

### Strengths and limitations

A major strength of this study is the high-quality national registries that made it possible to construct and validate the index in large populations. All patient contacts with the Norwegian health care system are recorded in these registries, except for a few private health services that operate outside the national health care system.

We harvested diagnoses for a period of three years (2015 − 2017) preceding the outcome in 2018. In terms of AUC this was clearly better than harvesting diagnoses only for 2017, and we recommend this approach. Increasing the observation time will give a more complete overview regarding morbidity.

Our aim was to develop an index suitable for registry data, solely based on ICPC-2 diagnostic codes as a predictor of mortality. One should be aware that such an index does not fully explain the magnitude of morbidity as a confounder, but indicates existence and direction [19]. Furthermore, the index does not describe multimorbidity or burden of disease. Consequently, large groups of patients comprised by the original list of conditions provided by Barnett *et al.* were not included in the ICPC morbidity index [15]. Although hypertension, coronary heart disease, atrial fibrillation, depression, anxiety and painful conditions contribute heavily to burden of disease in general practice populations, they have less influence on mortality than the conditions included in the ICPC morbidity index. Nevertheless, using this well-established multimorbidity list that has also been shown to predict mortality [16], is a strength regarding selection of diagnoses.

Some of the conditions listed in Table 1 had ORs significantly below 1. One could argue that some of these conditions should also be considered when constructing the index, not only those which were most positively associated with mortality. However, our aim was to construct an ICPC based mortality index that included the strongest predictors of death and that could be validated against the Charlson index, which is constructed in a similar way, not including “protective” conditions.

The original Charlson index included weights for disease severity [4], but such information is seldom available in registry-based materials [6]. Adding weights to the individual conditions in the ICPC morbidity index made little difference to its predictability. Therefore, we chose the non-weighted index.

The Charlson index was based on ICD-10 diagnostic codes from specialist health care, while the ICPC morbidity index was based on ICPC-2 diagnostic codes from primary care. Although the included diagnoses in the two indices partly overlap, it does not necessarily imply that the patients are the same. In contrast to the Charlson index we included diagnoses related to mental health and misuse of alcohol and other substances. This is probably the reason why the ICPC morbidity index had better predictive ability than the Charlson index in the younger age groups. Both indices had poorer predictive ability among older persons.

Ideally, external validation should be performed by other authors using a completely different population than the original study. Therefore, our strategy of using Norwegian born people for construction and foreign-born people for validation cannot be considered a true external validation, mainly because the doctors who coded the diagnoses were the same in the two materials.

### Findings in relations to other studies

The prevalence in Norway of most morbidities included in the ICPC morbidity index aligns well with other studies based on UK data and Read codes [15,16,20]. For some of the original morbidities it was not possible to find an ICPC-2 code that corresponded exactly with the Read code. In ICPC-2 it is not possible to distinguish between acute and chronic sinusitis. The most marked difference was found when prescriptions had been used as inclusion criteria. Our definitions of painful conditions and skin diseases were much broader than the UK data. However, these morbidities were not included in the final index.

To our knowledge this is the first attempt to develop an ICPC based morbidity index. In the UK several indices have been developed based on Read codes. Khan *et al.* translated the Charlson index for Read and OXMIS coded data used in the General Practice Research Database and found that the resulting index was a good predictor of mortality [8]. Another morbidity index based on Read codes developed by Carey *et al.* performed as well as the Charlson index [10].

The Cambridge Multimorbidity Score is also based on Read codes and the same list of morbidities as we used [15,16]. This score was tested with three different outcomes (primary care consultations, unplanned hospital admission and death) and performed better than the Charlson index. We found good alignment between the morbidities predicting mortality in the Cambridge score and our ICPC morbidity index. The most marked difference was found for painful conditions that had low OR in our initial analysis and was not included in the index. However, the Hazard ratio for this morbidity was 1.61 in the Cambridge score, reflecting the usefulness of including prescriptions to define more specific inclusion criteria for some conditions. The other differences were minor and related to morbidities that were not included the ICPC morbidity index.

Some studies have applied an age-adjusted version of the Charlson index by adding one point to the total score for each decade after the age of 50 years [21,22]. These studies have been based on hospital materials where the morbidity is higher, and weights for severity have been given to each diagnosis. Thereby, the unadjusted index will be far higher than what is present in our study. In our material age is a stronger predictor for mortality than both indices (Figure 1), and we believe it is more appropriate to use morbidity and age as two separate adjusting factors in future studies.

## Conclusion

We believe that the present ICPC morbidity index may be a useful tool for epidemiological research in primary care databases.
